# Reweighted Manifold Learning of Collective Variables from Enhanced Sampling Simulations

**DOI:** 10.1021/acs.jctc.2c00873

**Published:** 2022-11-11

**Authors:** Jakub Rydzewski, Ming Chen, Tushar K. Ghosh, Omar Valsson

**Affiliations:** † Institute of Physics, Faculty of Physics, Astronomy and Informatics, Nicolaus Copernicus University, Grudziadzka 5, 87-100 Toruń, Poland; ‡ Department of Chemistry, Purdue University, West Lafayette, Indiana 47907, United States; § Department of Chemistry, University of North Texas, Denton, Texas 76201, United States

## Abstract

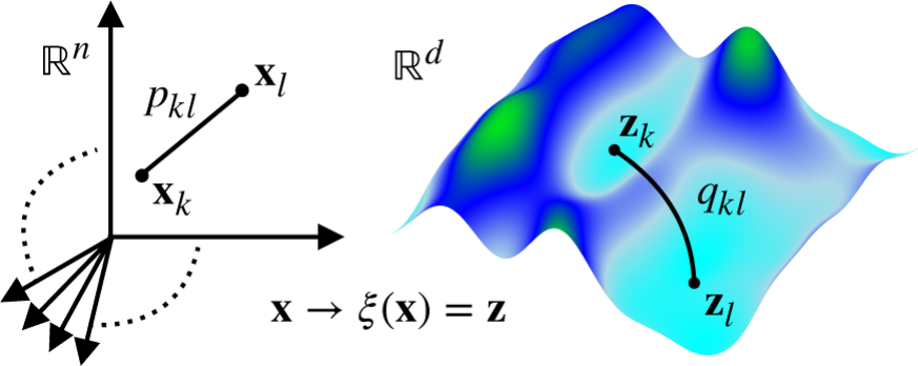

Enhanced sampling methods are indispensable in computational chemistry and physics, where atomistic simulations cannot exhaustively sample the high-dimensional configuration space of dynamical systems due to the sampling problem. A class of such enhanced sampling methods works by identifying a few slow degrees of freedom, termed collective variables (CVs), and enhancing the sampling along these CVs. Selecting CVs to analyze and drive the sampling is not trivial and often relies on chemical intuition. Despite routinely circumventing this issue using manifold learning to estimate CVs directly from standard simulations, such methods cannot provide mappings to a low-dimensional manifold from enhanced sampling simulations, as the geometry and density of the learned manifold are biased. Here, we address this crucial issue and provide a general reweighting framework based on anisotropic diffusion maps for manifold learning that takes into account that the learning data set is sampled from a biased probability distribution. We consider manifold learning methods based on constructing a Markov chain describing transition probabilities between high-dimensional samples. We show that our framework reverts the biasing effect, yielding CVs that correctly describe the equilibrium density. This advancement enables the construction of low-dimensional CVs using manifold learning directly from the data generated by enhanced sampling simulations. We call our framework reweighted manifold learning. We show that it can be used in many manifold learning techniques on data from both standard and enhanced sampling simulations.

## 1 Introduction

Among the main challenges in atomistic simulations of chemical systems is the significant temporal disparity between the timescales explored in standard atomistic simulations and the long timescales observed in experiments. Atomistic simulations can only reach timescales of up to milliseconds and thus cannot exhaustively sample the high-dimensional phase space, leading to the so-called sampling problem that has both theoretical and computational consequences for dynamical systems. The reason for the sampling problem is that these systems are characterized by many metastable states (i.e., high-probability regions) separated by energy barriers (i.e., low-probability regions) much higher than thermal energy (≫*k*_B_*T*). This leads to the kinetic entrapment of the system in a single metastable state as on the timescales obtained in standard atomistic simulations transitions to other metastable states are infrequent events. Such transitions between metastable states can be related to a few slow degrees of freedom that define a low-dimensional energy landscape. Examples of processes exhibiting metastability include catalysis,^1^ phase and glass transitions,^2−4^ photoactivation,^5,6^ and ligand dissociation.^7−10^

A possible resolution to the sampling problem is given by enhanced sampling methods.^11−15^ Over the years, various strategies for enhanced sampling have emerged, for example, tempering, variational, or biasing approaches; see ref (15) for classification and references therein. In this article, we consider a class of such enhanced sampling methods based on the work by Torrie and Valleau,^16^ which devised a framework for enhanced sampling that modifies the Boltzmann probability distribution by introducing a bias potential acting in a low-dimensional space of collective variables (CVs) that corresponds to slow degrees of freedom. However, identifying the reduced space of these CVs capturing the underlying chemical processes must be done before enhanced sampling simulations; it is far from trivial and often relies on experience and intuition. Consequently, many data-driven approaches are used to perform dimensionality reduction and construct CVs using samples directly from exploratory trajectories.^17−27^

An example of such data-driven approaches is manifold learning.^28^ The core of most manifold learning methods is having a notion of similarity between high-dimensional data samples, usually through a distance metric.^29−31^ The distances are integrated into a global parametrization of the data using kernels to represent a Markov chain containing information about transition probabilities that can be used to learn a smooth and low-dimensional manifold that captures the essentials of the data. This way, we can employ dimensionality reduction methods to learn CVs corresponding to slow degrees of freedom. We can distinguish two main approaches that manifold learning methods take to obtain a mapping to a low-dimensional representation of data: (i) eigendecomposition^32−40^ and (ii) divergence optimization.^30,31,41^

When using manifold learning on dynamical data resulting from atomistic simulations, these data must contain statistically sufficient information about the sampled chemical process. If a high-dimensional data set used in manifold learning does not capture the rare transitions between metastable states, the learned low-dimensional CVs will not capture them either. Unbiased atomistic simulations by construction sample only a fraction of the available configuration space and generally capture fast equilibrium processes. Therefore, employing unbiased simulations as learning data sets for manifold learning methods can lead to undersampled and nonoptimal CVs that do not capture the slow degrees of freedom corresponding to the rare chemical processes.

We can circumvent this issue by using the learning data set from enhanced sampling simulations, where transitions between metastable states are more frequently observed and are no longer rare events. However, in this case, the simulation data set is biased and does not correspond to the real system, as it is sampled from a biased probability distribution. Using these biased simulation data directly in manifold learning algorithms renders low-dimensional manifolds that are also biased (i.e., their geometry, density, and importance) and thus CVs that do not correspond to the chemical process. Therefore, in manifold learning, we need to correctly take into account that we use biased simulation data when learning CVs from enhanced sampling simulations. Despite several attempts in this direction,^22,24,42−46^ this area remains unexplored.

In this work, we consider the problem of using manifold learning methods on data from enhanced sampling simulations. We provide a unified framework for manifold learning to construct CVs using biased simulation data, which we call reweighted manifold learning. To this aim, we derive a pairwise reweighting procedure inspired by anisotropic diffusion maps, which accounts for sampling from a biased probability distribution. We term this procedure diffusion reweighting. Our framework considers the underlying geometry, density, and importance of the simulation data to construct a low-dimensional manifold for CVs encoding the most informative characteristics of high-dimensional dynamics of the atomistic system.

Our general framework can be used in many manifold learning techniques on data from both standard and enhanced sampling atomistic simulations. We show that our diffusion reweighting procedure can be employed in manifold learning methods that use both eigendecomposition and divergence optimization. We demonstrate the validity and relevance of our framework on both a simple model potential and high-dimensional atomistic systems.

## 2 Theory

In this section, we introduce the theory behind CVs (Section 2.1), enhanced sampling (Section 2.2), reweighting (Section 2.3), and biased data (Section 2.4) that we need to derive diffusion reweighting (Section 2.5).

### 2.1 Collective Variables

In statistical physics, we consider an *n*-dimensional system specified in complete detail by its configuration variables **x** ∈ *R*^*n*^. These configuration variables indicate the microscopic coordinates of the system or any other variables (i.e., functions of the microscopic coordinates) relative to the studied process, for example, an invariant representation. As a result, such a statistical representation is generally of high dimensionality.

In general, the configuration variables **x** are sampled during a simulation according to some, possibly unknown, high-dimensional probability distribution *P*(**x**) that has a corresponding energy landscape *U*(**x**) given by the negative logarithm of the probability distribution and an appropriate energy scale. If **x** consists of the microscopic coordinates, this distribution is known and is the stationary Boltzmann distribution
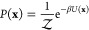
1where *U*(**x**) is the potential energy function of the system, the canonical partition function is , and β^–1^ = *k*_B_*T* is the thermal energy, with *T* and *k*_B_ denoting the temperature and Boltzmann’s constant, respectively. Without the loss of generality, we limit the discussion to the canonical ensemble (*NVT*) here.

The high-dimensional description of the system is very demanding to work with directly; hence, many classical approaches in statistical physics were proposed to introduce a coarse-grained representation, for example, the Mori–Zwanzig formalism^47,48^ or Koopman’s theory.^49^

To reduce the dimensionality of the high-dimensional space and obtain a more useful representation with a lower number of degrees of freedom, we map the configuration variables to a limited number of functions of the configuration variables, or so-called CVs. A corresponding target mapping ξ is the following
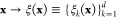
2where *d* is the number of CVs (*d* ≪ *n*) and {ξ_*k*_} are CVs.

**Figure 1:**
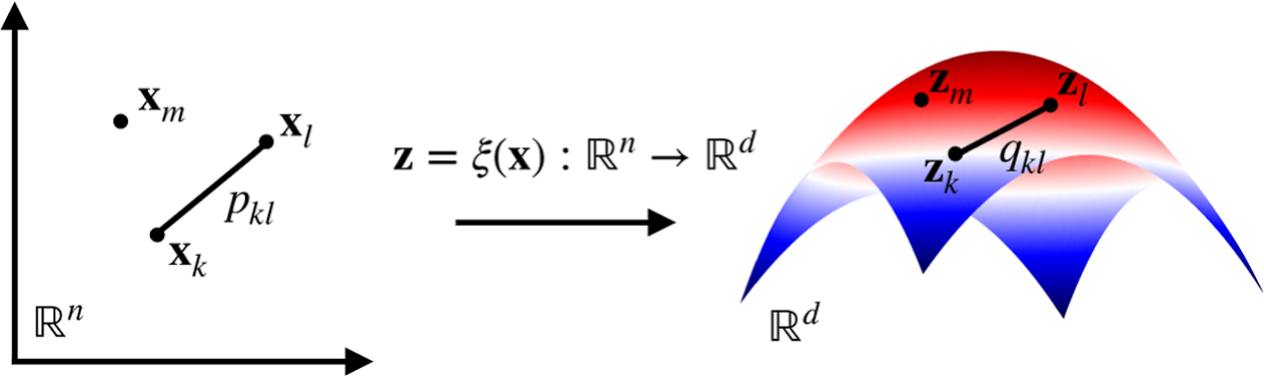
Target mapping from high-dimensional samples of configuration variables **x** to a low-dimensional manifold spanned by CVs **z**. In our framework, learning CVs is equivalent to finding the optimal parametrization of the target mapping **z** = ξ(**x**) [eq 2]. The target mapping performs the reduction from *R*^*n*^ to *R*^*d*^ so that the relation *p*_*kl*_ between the high-dimensional samples **x**_*k*_ and **x**_*l*_ is preserved in the relation *q*_*kl*_ in a low-dimensional manifold between the CV samples **z**_*k*_ and **z**_*l*_. For a detailed discussion, see Sections 2.5 and 3.2.

The parametrization of the target mapping is performed to retain the system characteristics after embedding into the low-dimensional CV space (Figure 1). In contrast to the configuration variables **x**, there are several requirements that the optimal CVs should fulfill, that is, (i) they should be few in number (i.e., the CV space should be low-dimensional), (ii) they should correspond to slow modes of the system, and (iii) they should separate relevant metastable states. If these requirements are met, we can quantitatively describe rare events.

Let us assume that the target mapping and the CVs are known. Then, we can calculate the equilibrium marginal distribution of CVs by integrating over other variables
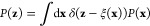
3where the δ-distribution is δ(**z** – ξ(**x**)) = ∏_*k*_δ(*z*_*k*_ – ξ_*k*_(**x**)).

Having the marginal equilibrium probability, we can define the free-energy landscape in the CV space as the negative logarithm multiplied by the thermal energy
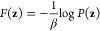
4

In practice, free-energy landscapes for systems severely affected by the sampling problem are characterized by many metastable states separated by high kinetic barriers that impede transitions between metastable states. Consequently, on the timescales that we can simulate, the system stays kinetically trapped in a single free-energy minimum and cannot explore the CV space efficiently.

### 2.2 Enhanced Sampling

CV-based enhanced sampling techniques overcome the sampling problem by introducing a bias potential *V*(**z**) acting in the CV space designed to enhance CV fluctuations. The functional form of the bias depends on the enhanced sampling method used.^12,15,16,50−52^ The bias potential can be static^16^ or adaptively constructed on the fly during the simulation.^12,15,50−52^ Regardless of how the bias potential is constructed, it leads to a biased CV distribution that is smoother and easier to sample than the unbiased distribution [eq 3]
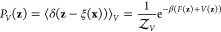
5where ⟨·⟩_*V*_ denotes the biased ensemble average, and the biased partition function is .

CV-based enhanced sampling methods construct the bias potential to reduce or entirely flatten free-energy barriers. Let us consider well-tempered metadynamics,^51^ which is the method we employ in this work. Well-tempered metadynamics uses a history-dependent bias potential updated iteratively by periodically depositing Gaussians centered at the current location in the CV space. The bias potential is given as
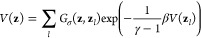
6where *G*_σ_(**z**, **z**_*l*_) is a scaled Gaussian kernel with a bandwidth set σ, **z**_*l*_ is the center of *l*-th added Gaussian, and γ is a bias factor that determines how much we enhance CV fluctuations. Well-tempered metadynamics convergences to a biased CV distribution given by the so-called well-tempered distribution
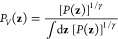
7which we can view as sampling an effective free-energy landscape *F*/γ with barriers reduced by a factor of γ.

### 2.3 Reweighting

Biasing results in a gradual divergence from the equilibrium CV distribution to a smoother and easier to sample biased CV distribution, that is, from eq 3 to eq 7 in the case of well-tempered metadynamics. Consequently, the importance of each sample is given by a statistical weight needed to account for the effect of the bias potential when obtaining equilibrium properties such as the free-energy landscape. This contrasts with unbiased simulations where samples are equally important as they are sampled according to the equilibrium distribution.

A functional form of the weights depends on a particular method. Generally, for methods employing a bias potential *V*(**z**), the weight associated with a CV sample **z** can be written as
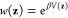
8In the case of a static bias, the weights are given by eq 8. In contrast, well-tempered metadynamics uses an adaptive bias potential [eq 6], and we need to account for a time-dependent constant given by^12,53^
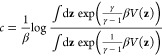
9which is independent of **z**. We can then redefine the weights as
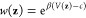
10where *V*(**z**) − *c* is called the relative bias potential.

Note that in the abovementioned discussion, we assume that the dependence of the bias potential on the simulation time is implicit. We can ignore the time dependence once the simulation reaches convergence; then, the relative bias potential *V*(**z**) − *c* is quasi-stationary and does not change considerably (the bias potential *V*(**z**) and the time-dependent constant *c* can still increase, while the relative bias potential converges). In practice, when performing reweighting, we ignore a short initial transient part of the simulation, where the relative bias potential is still changing considerably.

The standard reweighting works by employing the weights to obtain the stationary equilibrium distribution from the biased CV distribution, that is, *P*(**z**) ∝ *w*(**z**)*P*_*V*_(**z**). The unbiased probability distribution *P*(**z**) can be computed by histograming or kernel density estimation, where each sample **z** is weighted by eq 8. This is done routinely in advanced simulation codes, for example, plumed.^54,55^

Manifold learning methods cannot use the standard reweighting to unbias pairwise relations between samples. Instead, a nontrivial approach to reweighting in the form of *r*(**x**_*k*_, **x**_*l*_) is required, where *r*(**x**_*k*_, **x**_*l*_) is a pairwise reweighting factor that characterizes the importance of the relation between samples **x**_*k*_ and **x**_*l*_.

### 2.4 Biased Data for Manifold Learning

Given the requirements for the optimal CVs (Section 2.1), it is nontrivial to provide low-dimensional CVs knowing only the microscopic coordinates. Instead, we often resort to an intermediate description and select a large set of the configuration variables (often called features). For example, this might be internal coordinates such as distances, dihedral angles, and so forth. These configuration variables then define a high-dimensional space, which we reduce to the optimal low-dimensional CVs. For a list of helpful configuration variables to characterize different chemical systems, see, for example, the plumed documentation.^56^

Consider data obtained from enhanced sampling simulations in which we record or select samples of the high-dimensional configuration variables **x**. These data define the training set from which manifold learning methods construct a low-dimensional manifold. The training data set can be generally expressed as
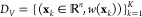
11where *K* is the number of samples, and the sample set is augmented by the corresponding statistical weights. Note that the weights depend on **x** through CV mapping [eq 2].

### 2.5 Diffusion Reweighting

Geometrically, the existence of a low-dimensional representation assumes that the high-dimensional dynamical system populates a low-dimensional manifold. This assumption is known as the manifold hypothesis.^42^ Under this view, the fast degrees of freedom are adiabatically slaved to the dynamics of the slow degrees of freedom, which correspond to the optimal CVs due to the presence of fast equilibration within the metastable states. Methods leveraging this assumption belong to a class of manifold learning techniques.

The core of manifold learning methods appropriate for dimensionality reduction in dynamical systems is the construction of a random walk through a Markov chain on the data set, where the transition probabilities *p*_*kl*_ depend on a kernel function and distances between samples. Depending on how the transition probabilities *p*_*kl*_ are used to find a target mapping to a low-dimensional manifold, we can distinguish two main approaches: (i) eigendecomposition^32−40^ and (ii) divergence optimization.^30,31,41^ In manifold learning methods using eigendecomposition, eigenvalues and eigenvectors are used to construct the target mapping. In methods employing divergence optimization, however, the transition probabilities *p*_*kl*_ are used to find a Markov transition matrix *q*_*kl*_ constructed from low-dimensional samples (Figure 1).

Although many kernels can be considered in manifold learning, a typical choice in spectral embedding methods is a Gaussian kernel dependent on Euclidean distances^29,36^
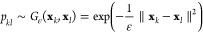
12where ε is a positive parameter chosen depending on the given data set, as it induces a length scale  that should match the distance between neighboring samples. Equation 12 models the Markov transition matrix if every row is normalized to unity.

However, this construction includes information only on the manifold geometry given by the pairwise distances. The remaining components required for our reweighting approach are the density and importance of the data.

For the Markov transition matrix, the reweighting procedure must be reformulated to include the weights *w*(**x**_*k*_) and *w*(**x**_*l*_) for a pair of samples **x**_*k*_ and **x**_*l*_, respectively. Our plan is to derive such a pairwise reweighting formula, where each pairwise transition probability given by the Markov transition matrix *M*(**x**_*k*_, **x**_*l*_) depends also on a reweighting factor *r*(**x**_*k*_, **x**_*l*_). We assume that a reweighted Markov transition matrix can be defined in a simple form
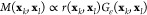
13where *M* is row-stochastic. The Markov transition matrix then models the unbiased Markov chain, where each entry is the probability of the jump from **x**_*k*_ to **x**_*l*_.

To account for the manifold density, we need to employ a density-preserving kernel. In contrast to Laplacian eigenmaps that are appropriate for data sampled uniformly,^29,35^ diffusion maps allow working with data sampled from any underlying probability distribution. Specifically, let us consider the pairwise transition probabilities based on an anisotropic diffusion kernel given by^36^
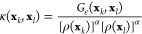
14where ρ(**x**) is a kernel density estimator and α ∈ [0, 1] is the anisotropic diffusion parameter, which is crucial to properly include information about the data density and importance.^37^ Based on the anisotropic diffusion parameter, a diffusion map can be used to parametrize a family of low-dimensional embeddings.

In eq 14, the density estimator ρ(**x**_*k*_) at a sample **x**_*k*_ must be reweighted to account on the data importance
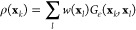
15which is a weighed kernel density estimate up to an unimportant multiplicative constant. After the reweighting, the density estimator characterizes the unbiased density, in contrast to the biased density estimate that is given as
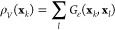
16where the subscript *V* denotes that the density estimate is calculated under the bias potential *V*.

In theory, if the underlying probability distribution of high-dimensional samples is known analytically, it is possible to express ρ directly from this distribution;^39^ for example, from a Boltzmann distribution [eq 1] if the samples are represented by the microscopic coordinates. However, this is valid only in the case of sufficient sampling and is thus rarely reachable in practice. Moreover, the high-dimensional distribution *P*(**x**) of the configuration variables is unknown in general (Section 2.1). For this reason, we write ρ as a kernel density estimate [eq 15].

We can understand the meaning behind the anisotropic diffusion kernel by considering eq 14. The dynamics described by eq 14 is local as samples closer to each other have a higher probability of being close in the respective low-dimensional manifold and vice versa in the case that they are farther apart. This information about the underlying geometry is given by *G*_ε_(**x**_*k*_, **x**_*l*_), which requires that the transition probabilities are penalized between the geometrically distant samples **x**_*k*_ and **x**_*l*_. The density and importance of samples are encoded in the unbiased density estimates [eq 15].

Depending on the α value in eq 14, three interesting cases of diffusion maps can be considered asymptotically.^37^ Namely, (i) for α = 1/2, eq 14 corresponds to the Markov chain that is an approximation of the diffusion given by the Fokker–Planck generator, with the underlying data density proportional to the equilibrium density, allowing us to approximate the long-time behavior of the microscopic coordinates. Other values of α are also possible, for example, (ii) for α = 0, we get the classical normalized graph Laplacian, and (iii) for α = 1, we ignore the underlying density, and the diffusion operator approximates the Laplace–Beltrami operator. We note that this asymptotic behavior holds in the limit of infinite data *K* → ∞ and ε → 0 when considering the microscopic coordinates. As we are interested in finding low-dimensional CVs, the case for α = 1/2 is appropriate to model asymptotically the slowest degrees of freedom, accounting for both the underlying geometry and density of the manifold.

As we have all the required ingredients for the reweighting of Markov transition matrices, we focus on deriving the reweighting factor. Here, we discuss only an outline, while a detailed derivation is provided in Appendix A.

Based on eq 14, the Markov transition matrix can be estimated by weighting each Gaussian term and normalizing it so that it is row-stochastic
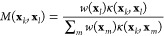
17

Next, by inserting eq 14 to eq 17, we can see that the Markov transition matrix *M* can be written also using the Gaussian kernels
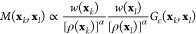
18where we can recognize the reweighting factor by comparing the result to eq 13. Therefore, we get the following expression
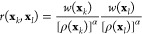
19

We can also approximate the reweighting factor by rewriting eq 19 with the biased density estimate [eq 16]
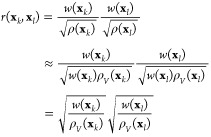
20where we set α = 1/2. Equation 20 is the final form of the reweighting factor that we use here. A detailed derivation of eq 20 is provided in Appendix A. Although the derivation of eq 20 is presented using the Gaussian kernel, our framework can be used in other manifold learning methods, as demonstrated in Section 3.

Equation 18 denotes an unbiased Markov chain with the transition probability from **x**_*k*_ to **x**_*l*_ in one time step *t* given by
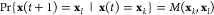
21

We term our reweighting procedure diffusion reweighting. We postulate that the derived Markov transition matrix [eq 18] has the following three properties that make the construction of eq 21 from enhanced sampling simulations feasible. Namely, the Markov transition matrix encodes the information about:

1 Geometry *G*_ε_(**x**_*k*_, **x**_*l*_): The probability of transitions between samples lying far from each other is low, and it is high for those in close proximity.
2 Density : The anisotropic diffusion constant α ∈ [0, 1] is used as a density-scaling term, as in diffusion maps. See eq 14 and the corresponding description.
3 Importance *w*(**x**_*l*_): The statistical weights from enhanced sampling decide according to the bias if a sample is important, that is, the metastable states where the weights are higher are more important than high free-energy regions.

### 2.6 Implementation

Our framework is implemented in a development version of plumed 2.7^54,55^ as the LowLearner module and will be made publicly available in the coming future. Its initial implementation incorporating several algorithms used in this work can be accessed at Zenodo (doi: 10.5281/zenodo.4756093) and from plumed-nest(55) repository under plumID:21.023 at https://plumed-nest.org/eggs/21/023/.

## 3 Reweighted Manifold Learning

We incorporate diffusion reweighting into several manifold learning methods and apply them to find a low-dimensional representation in a model system and high-dimensional atomistic simulation problems represented by biased simulation data. Specifically, we consider diffusion reweighting in diffusion maps^37−39^ and recently introduced stochastic embedding methods for learning CVs and adaptive biasing.^22,24^

To demonstrate the validity of our framework, we construct a diffusion map for standard testing systems such as a particle moving on an analytical potential and alanine dipeptide. For the stochastic embedding methods, we choose a mini-protein chignolin. For the two atomistic systems, alanine dipeptide and chignolin, we describe the systems using two different types of high-dimensional representations (distances and dihedral angles, respectively) to show that the framework can work regardless of the chosen configuration variables.

### 3.1 Diffusion Maps

We start by considering the case of diffusion maps, on which we base the derivation of the reweighting factor *r*(**x**_*k*_, **x**_*l*_) (Section 2.5). By rewriting the diffusion kernel using the biased density estimates [eq 20], we can use it to construct a low-dimensional embedding from a biased data set. We directly use eq 18 to estimate the transition probabilities, while using eq 20, to account for the sampling from any biased distribution.

#### 3.1.1 Target Mapping ξ(**x**): Eigendecomposition

With the exemption of the reweighting factor, further steps in our approach to diffusion maps proceed as in its standard formulation.^37^ Let us briefly recap these steps.

In diffusion maps, the spectral decomposition of the Markov transition matrix *M* is performed to define a low-dimensional embedding, *M*ψ = λψ, where {λ_*l*_} and {ψ_*l*_} are eigenvalues and eigenvectors, respectively. The eigenvalues are related to the effective timescales as  and can be used to determine the slowest processes in the dynamics. Then, the eigenvectors corresponding to the largest eigenvalues define a reduced space. Given this interpretation, the target mapping [eq 2] is defined by the diffusion coordinates
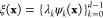
22where ξ(**x**) is computed using the first *d* eigenvalues and eigenvectors, with the equilibrium density represented by the zeroth coordinate λ_0_ψ_0_. In eq 22, the spectrum of the eigenvalues {λ_*l*_} is sorted by the nonincreasing value, λ_0_ = 1 > λ_1_ ≥··· ≥ λ_*d*–1_.

The truncation up to *d* – 1 of eq 22 for metastable systems corresponds to a negligible error on the order of *O*(λ_*d*_/λ_*d*–1_).^37^ In other words, this assumption relates to a large spectral gap that separates slow degrees of freedom  and fast degrees of freedom . For a detailed description behind the construction of the diffusion coordinates from unbiased data, we refer to works by Coifman.^36,38,39^ By inspecting the spectral gap obtained via the eigendecomposition of the reweighted Markov transition matrix, it is possible to verify that the selected high-dimensional representation sampled from a biased distribution contains enough information to render a physically meaningful low-dimensional manifold.
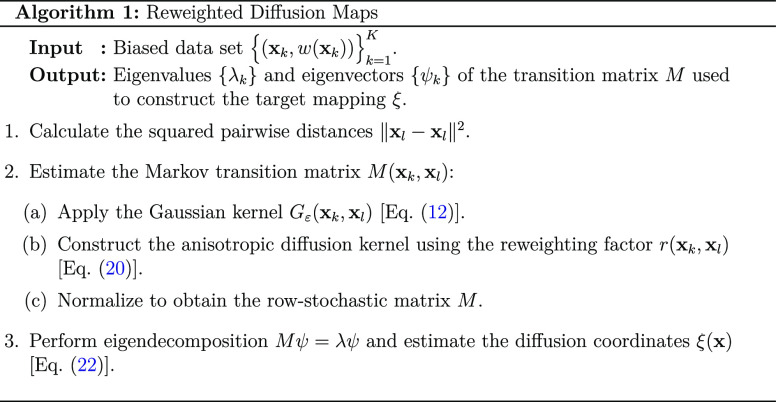


#### 3.1.2 Example: Model Potential

**Figure 2:**
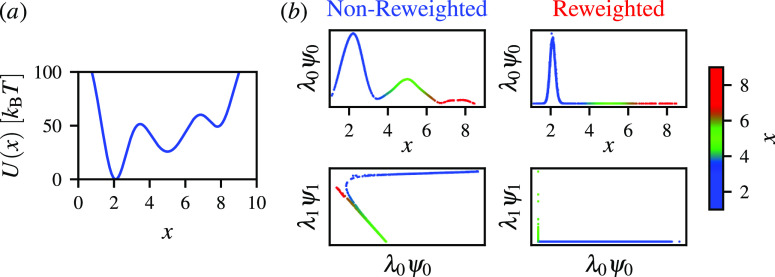
Diffusion maps generated for the reweighted and nonreweighted (without applying diffusion reweighting) biased simulation of a particle in simple (a) one-dimensional potential *U*(*x*), where the energy barriers separating the deepest minimum are on the order of 50 *k*_B_*T*, and the corresponding transitions from this state are rare events. (b) Comparison between the nonreweighted (blue) and reweighted (red) diffusion maps: the equilibrium densities along the coordinate *x* and diffusion coordinates λ_0_ψ_0_*vs* λ_1_ψ_1_, with coloring according to the *x* value. The enhanced sampling simulation is performed using well-tempered metadynamics^51^ with a bias factor of 10 by employing the pesmd code in the plumed(54,55) plugin.

As a simple and illustrative example of applying diffusion reweighting within the diffusion map framework, we consider a case where dimensionality reduction is not performed. Namely, we run an enhanced sampling simulation of a single particle moving along the *x* variable on a one-dimensional potential *U*(*x*) with three Gaussian-like metastable states with different energy depths and energy barriers between the minima [Figure 2a]. In this system, the highest energy barrier is ∼50 *k*_B_*T*, which makes the transitions from the deepest minimum rare. The dynamics is modeled by a Langevin integrator^57^ using temperature *T* = 1, a friction coefficient of 10, and a time step of 0.005. We employ the pesmd code in the plumed(54,55) plugin. We bias the *x* variable using well-tempered metadynamics^51^ with a bias factor of γ = 10. Further details about the simulation are given in Supporting Information in Section S1A.

We present our results in Figure 2b. We can see that the nonreweighted (without applying diffusion reweighting) diffusion map learns the biased distribution (given by λ_0_ψ_0_) along the coordinate *x*, where the three energy minima correspond to the maxima of the biased distribution. Additionally, the first two diffusion coordinates are not orthogonal, and there is a lack of separation between the metastable states.

In contrast, the reweighted diffusion map can represent the equilibrium density (λ_0_ψ_0_), where only the first energy minimum is populated due to the high-free energies separating the states. The λ_0_ψ_0_ and λ_1_ψ_1_ diffusion coordinates properly separate the samples. We can see that λ_1_ψ_1_ is almost marginal due to the lack of additional dimensions for the potential energy.

The example presented in Figure 2 is, of course, a trivial case in which no dimensionality reduction is performed; however, it indicates that diffusion reweighting can be used to reweight the transition probabilities successfully and that the standard diffusion map trained on the biased data captures an incorrect representation.

#### 3.1.3 Example: Alanine Dipeptide

As a next example, we consider alanine dipeptide (Ace-Ala-Nme) in a gas phase described using the Amber99-SB force field.^58^ The data set is generated by a 100 ns molecular dynamics simulation^59,60^ using the gromacs 2019.2 code^61^ patched with a development version of the plumed(54,55) plugin. The simulation is performed by well-tempered metadynamics^51^ at 300 K using the backbone dihedral angles Φ and Ψ for biasing with a bias factor of 5. We select the Φ and Ψ dihedral angles as biasing them is sufficient to sample accelerated transitions between several metastable states of alanine dipeptide. Using this setup, the convergence of the bias potential is obtained quickly. Further details about the simulation are given in Supporting Information (Section S1B).

Using diffusion maps, we reduce the high-dimensional space consisting of all pairwise distances between the heavy atoms (*n* = 45) to two dimensions. The diffusion maps are constructed using ε = 0.078 estimated as the median of the pairwise distances.

**Figure 3:**
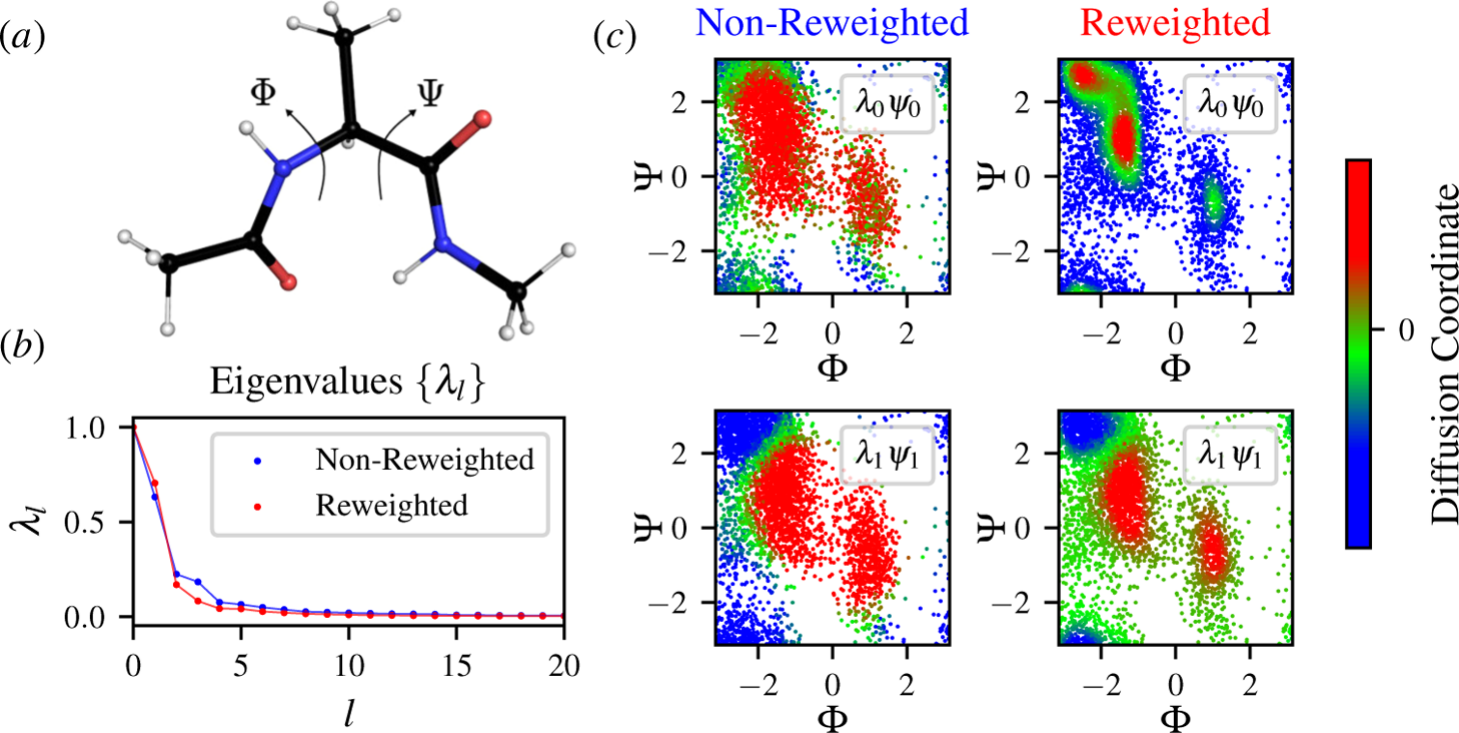
Reweighted diffusion maps on a peptide model system (Ace-Ala-Nme) in vacuum at 300 K simulated using well-tempered metadynamics^51^ enhancing the Φ and Ψ dihedral angles and a bias factor γ = 5. The diffusion map is calculated using a high-dimensional space of 45 pairwise distances between heavy atoms. (a) Representative structure of alanine dipeptide with the dihedral angles Φ and Ψ. (b) Spectrum of eigenvalues {λ_*l*_} obtained from the eigendecomposition for the nonreweighted (blue) and reweighted (red) Markov transition matrices. (c) Samples are shown in the dihedral angle space for the nonreweighted (blue label) and reweighted (red label) diffusion map with colors representing the first and second diffusion-map coordinates λ_0_ψ_0_(**x**) and λ_1_ψ_1_(**x**), respectively. The color bar represents the constructed diffusion coordinates.

We present diffusion reweighting results for alanine dipeptide in Figure 3. The eigenvalues of the Markov transition matrix have a spectral gap (i.e., timescale separation) with only a few eigenvalues close to one and all other eigenvalues much smaller than one. Thus, only the first few eigenvectors are needed to approximate the diffusion coordinates [eq 22], and thus the target mapping to the CV space. The eigenvalues {λ_*l*_} indicate that the spectral gap is slightly wider for the reweighted transition probability matrix, as can be seen in Figure 3b. Consequently, the effective timescales  calculated from the eigenvalues indicate that the reweighted diffusion map corresponds to slower processes; see Supporting Information (Figures S3 and S4).

We can see that the nonreweighted approach cannot correctly account for the transition probabilities calculated based on the biased simulation, as we expected. The transitions between the metastable states are so frequent that the zeroth diffusion coordinate (the equilibrium density) suggests only one metastable state [Figure 3c]. In Supporting Information (Figure S2), we show that the separation of samples in the reweighted diffusion map is much better than for the nonreweighted diffusion map. It resembles a “typical” diffusion map from unbiased data sets.

In the reweighted case, the low-dimensional coordinates can distinguish between the relevant metastable states. Additionally, using eq 20, the zeroth diffusion-map coordinate, λ_0_ψ_0_(**x**), correctly encodes the information about the Boltzmann equilibrium distribution of alanine dipeptide in the dihedral angle space, which is not possible using the standard (i.e., nonreweighted) diffusion map in the case of biased simulation data [Figure 3c]. By comparing the reweighted diffusion map to a diffusion map constructed from an unbiased parallel tempering replica at 300 K, we can see that the embeddings and eigenvalues are virtually identical; see Supporting Information (Figure S5).

These results further corroborate our findings and show that when performing a dimensionality reduction from data resulting from enhanced sampling, the reweighting factor [eq 20] is needed to revert the effect of biasing in the transition probability matrix.

### 3.2 Stochastic Embeddings

Next, we move to employ diffusion reweighting in more recent approaches. We consider manifold learning methods devised primarily to learn CVs from biased simulation trajectories: multiscale reweighted stochastic embedding (mrse)^24^ and stochastic kinetic embedding (stke).^22^ These methods use approximations of the reweighting factor [eq 20]. Our aim is not to compare results obtained using these methods but to present and discuss how diffusion reweighting can be approximated and employed in manifold learning methods other than diffusion maps.

First, let us focus on a general procedure these stochastic embedding methods use to parametrize manifolds. Mainly, we discuss how these methods use the Markov transition matrices to parametrize the target mapping to low-dimensional manifolds. The construction of the Markov transition matrix with reweighting from biased data in each technique is discussed in the remainder of this section.

#### 3.2.1 Target Mapping ξ_θ_(**x**): Divergence Optimization

As mentioned above, the stochastic embedding methods belong to the second category of manifold learning methods we consider here, that is, based on divergence optimization. Thus, unlike diffusion maps, the eigendecomposition is not performed in these methods. Instead, the target mapping ξ is parametrized based on neural networks that perform nonlinear dimensionality reduction. The target mapping is given as
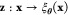
23where **θ** = {θ_*k*_} are parameters of the target mapping adjusted such that the low-dimensional manifold of CVs is optimal with respect to a selected statistical measure. Using eq 23, the distance between samples in a manifold can be given as
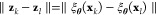
24

Note that in some simple cases, the mapping in eq 23 can also be represented using a linear combination. However, deep learning has been successful in a broad range of learning problems, and using more intricate approximations for the mapping between high-dimensional and low-dimensional spaces is quite common for complex data sets.^62,63^

The target mapping is parametrized by comparing the Markov transition matrix *M*(**x**_*k*_, **x**_*l*_) = (*p*_*kl*_) (Section 3.2.2) constructed from the high-dimensional samples to a Markov transition matrix *Q*(**z**_*k*_, **z**_*l*_) = (*q*_*kl*_) built from low-dimensional samples mapped using the target mapping [eq 23].

In stke, we use a Gaussian kernel for *Q*
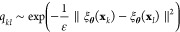
25

In mrse, we employ a one-dimensional *t*-distribution, as implemented in *t*-sne.^31,63^ Taking the target mapping as defined in eq 23, the transition probabilities in the low-dimensional space *q*_*kl*_ in mrse are
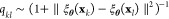
26

The choice of the *t*-distribution for *Q* in mrse is motivated by the apparent crowding problem,^31^ that is, as the volume of a small-dimensional neighborhood grows slower than the volume of a high-dimensional one, the neighborhood is stretched so that moderately distant sample pairs are placed too far apart. As outlined in ref (31), the use of a heavy-tailed distribution for the low-dimensional representation allows moderate distances in the high-dimensional space to be represented by much larger distances in the manifold, encouraging gaps to form in the low-dimensional map between the clusters present in the data, alleviating the crowding problem to some degree.

Finally, the Markov transition matrices computed from the high-dimensional and low-dimensional samples need to be compared. The most common choice for such a metric is employing a statistic distance, particularly the Kullback–Leibler divergence
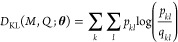
27where in contrast to the standard formulation of the Kullback–Leibler divergence that compares two probability distributions, eq 27 is computed for every pair of rows from *M* and *Q*, and then summed. Equivalently, we can minimize the cross-entropy,
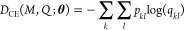
28as the probabilities *p*_*kl*_ stay constant during the optimization. There are many choices possible for the comparison between *M* and *Q*, for example, the Jensen–Shannon divergence.^22^

The Kullback–Leibler divergence optimization is performed to train the target mapping represented by a neural network. As the target mapping is parametric, the gradients of *D*_KL_, with respect to the parameters **θ** = {θ_*k*_} of the neural network, can be estimated effortlessly using backpropagation. For further details about training neural networks, we refer to Appendix E.

#### 3.2.2 Reweighted Markov Transitions

After explaining how the parametric mapping is constructed in the reweighted stochastic embeddings, we proceed to formulate the Markov transition matrices and the reweighting factors for these methods.

First, let us consider the reweighting performed in mrse.^24^ This method employs the following reweighting factor
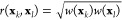
29where we neglect the biased density estimates ρ_*V*_ [cf. eqs 29 and 20]. The reweighting factor [eq 29] written as a geometric mean between two statistical weights can be justified by the fact that the bias potential is additive, as shown in eq 8, and a geometric mean is appropriate to preserve this relation. We note that similar reweighting procedures have been used in refs (45)(46), and (64).

The Markov transition matrix in mrse is expressed as a Gaussian mixture, where each Gaussian is evaluated for different ε values and reweighted using eq 29
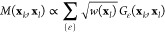
30where we omit the normalization constant for brevity. The sum in eq 30 is over bandwidths that are automatically estimated and selected to fit that data. Note that many methods can be used for this purpose; however, to facilitate analysis, we use a method from ref (24). As this procedure is mostly technical, for details about estimating bandwidths and constructing the Gaussian mixture, we refer to Appendix C.

Second, let us consider stke. Suppose high-dimensional samples are resampled so that each sample keeps a certain distance away from the others. In that case, the distribution of samples can be viewed as approximately uniform. Then, *w*(**x**) can be replaced by the unbiased probability density estimator ρ(**x**) in eq 29. Thus, the reweighting factor is given by
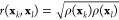
31which is the formula used in stke.^22,65^ The corresponding Markov transition matrix is
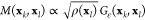
32where, as in eq 30, the *k*-th reweighting term is canceled out during normalization.

An interesting property of the transition probabilities used by this method is that by taking an approximation to the normalization constant (Appendix B), we arrive at a transition probability matrix of a similar form as in the square-root approximation of the infinitesimal generator of the Fokker–Planck operator^66−69^
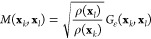
33for a single ε. The square-root approximation has been initially derived by discretizing a one-dimensional Smoluchowski equation.^70^ It can also be shown that eq 33 can be obtained using the maximum path entropy approach.^71,72^

As many algorithmic choices are available for each procedure incorporated in the reweighted stochastic embedding framework, it is difficult to directly compare mrse and stke. However, we aim to discuss how approximations of the reweighting factor are employed in these methods and how they can be used to learn CVs from biased data. Thus, in the abovementioned discussion, we focus on the reweighting procedures for the Markov transition matrices used by these methods. To compare the parameters used by these methods, see Appendix E.
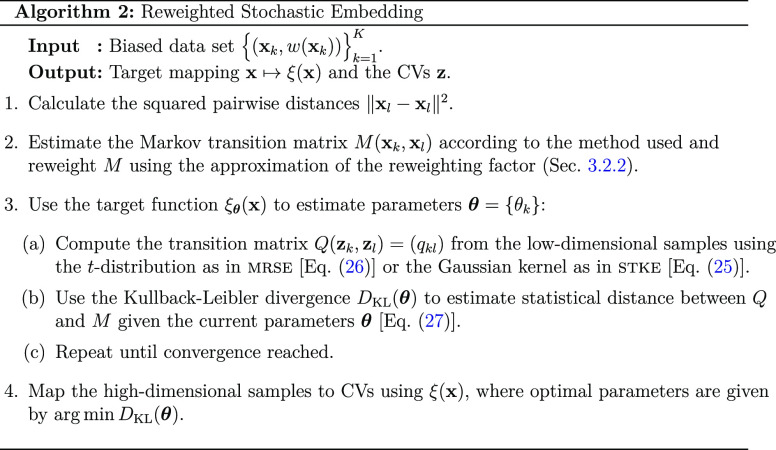


#### 3.2.4 Example: Chignolin

As an example for the two stochastic embedding methods, mrse and stke, we consider the folding and unfolding of a ten amino-acid miniprotein, chignolin (CLN025),^73^ in the solvent. We employ the CHARMM27 force field^74^ and the TIP3P water model,^75^ and we perform the molecular dynamics simulation^59,60^ using the gromacs 2019.2 code^61^ patched with a development version of the plumed(54,55) plugin. Our simulations are performed at 340 K for easy comparison with other simulation data, also simulated at 340 K.^76,77^ We perform a 1 μs well-tempered metadynamics simulation with a large bias factor of 20. We select a high bias factor to illustrate that our framework is able to learn metastable states in a low-dimensional manifold even when the free-energy barriers are virtually flattened and the system dynamics is close to diffusive at convergence.

**Figure 4:**
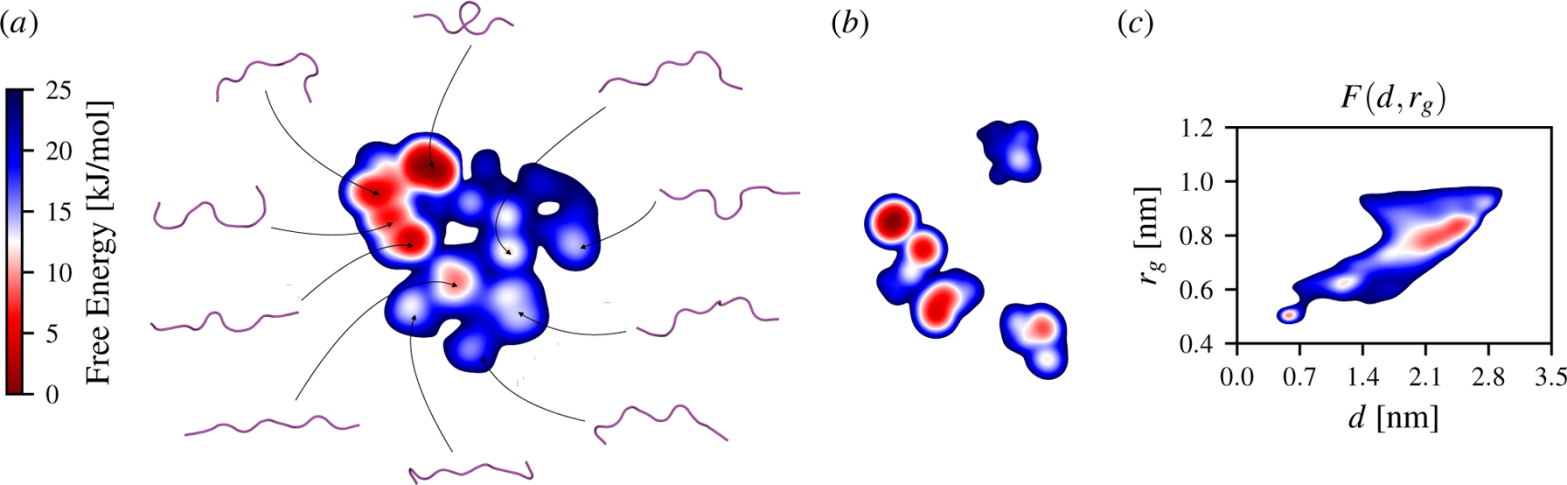
Reweighted stochastic embeddings calculated for chignolin in the TIP3P solvent at 340 K simulated using the CHARMM27 force field. Low-dimensional manifolds are colored according to their free energy. (a) Representative conformations from the metastable states estimated by the reweighted embedding methods are shown around the mrse embedding. (b) Embedding obtained using stke. Well-tempered metadynamics is used to generate the training set consisting of sines and cosines of all Φ and Ψ dihedral angles, amounting to 32 variables in total. The training set is generated by performing a 1 μs simulation with a bias factor γ = 20, enhancing the fluctuations of the distance *d* between the Cα atoms of residues Y1 and Y10 and the radius of gyration *r*_g_. (c) Free-energy surface calculated along for *d* and *r*_g_. The axes and units for the embeddings are arbitrary and thus not shown. See Supporting Information (Section S1C) for computational details.

As biased CVs, to enhance transitions between the folded and unfolded conformations of CLN025 in the metadynamics simulation, we choose the distance between Cα atoms of residues Y1 and Y10 (*d*) and the radius of gyration (*r*_g_) [Figure 4c]. We consider CLN025 conformations folded when the distance is below ∼0.8 nm and unfolded otherwise for >0.8 nm. From the resulting trajectory, we calculate the sines and cosines of all the backbone Φ and Ψ dihedral angles and use them as the high-dimensional representation of CLN025, which amounts to 32 variables in total. We collect high-dimensional samples every 1 ps for the biased training data set. Then, the low-dimensional manifolds are trained on representative samples selected, as described in refs (22) and (24). As we focus mainly on the Markov transition matrices and diffusion reweighting here, we provide a detailed discussion of the subsampling procedures in Appendix D.

In Figure 4, we present the resulting manifolds spanned by the trained CVs computed using the reweighted stochastic embedding methods (Section 3.2). The embedding presented in Figure 4a is calculated using mrse,^24^ while the embedding presented in Figure 4b is calculated using stke,^22^ using their corresponding reweighting formulas given by eqs 29 and 31, respectively. For each manifold, the corresponding free-energy landscapes are calculated using kernel density estimation using the weights to reweight each sample [eq 10].

We can observe that the free-energy landscape in the low-dimensional manifold calculated by mrse is highly heterogeneous, with multiple partially unfolded intermediate states and many possible reaction pathways, as shown in Figure 4a. Such a complex free-energy landscape shows that the dynamics of CLN025 is more intricate and complex than what is visible in the free-energy surface spanned by the distance and the radius of gyration [Figure 4c], where we can see only the folded, intermediate, and unfolded states and the remaining are possibly degenerate.

In Figure 4, we can see the lower-lying free-energy basins in the reweighted stochastic embeddings are captured by both mrse and stke. We can also notice a slight difference between the metastable states lying higher in free energy. Specifically, mrse captures more states below a threshold of 25 kJ/mol in comparison to the embedding rendered by stke, in which the rest of the states are placed over 25 kJ/mol (i.e., mainly different unfolded states).

In our simulations, we do not observe a misfolded state of CLN025 shown to be highly populated in several studies^78,79^ employing different force fields (Amber99^80^ and Amber99-SB,^58^ respectively) compared to CHARMM27 here.^74^ This misfolded state is also not observed in the long unbiased simulation from ref (76) that employs the same CHARMM27 force field as we do.

By comparing the free-energy barriers between the different embeddings in Figure 4, we can see that they are similar, particularly for the mrse embedding and the free-energy surface spanned by the distance and the radius of gyration, that is, from 10 to 15 kJ/mol. We can compare our results to the unbiased simulation data from the study of Lindorff-Larsen et al.,^76^ where the authors perform a very long simulation and observe a significant number of folding and unfolding events, thus allowing unbiased estimates of free-energy barriers to be obtained. In their study, CLN025 was shown to be a “fast folder” with a corresponding free-energy barrier of ∼10 kJ/mol. Similar estimates have also been obtained in ref (77). Therefore, we can conclude that the free-energy barriers in the embeddings agree well with previous computational studies.

Note that the simulation of CLN025 performed in ref (76) is ∼100 μs long, compared to our 1 μs simulation. This clearly illustrates the great benefit of combining manifold learning with the ability to learn from biased data sets.

Overall, both the separation of the CLN025 metastable states and the free-energy landscapes calculated for the low-dimensional embeddings suggest that the proposed framework can be used to find slow CVs and physically valid free-energy estimates. The presented results (Figure 4) clearly show that using our approach, we can construct a meaningful and informative low-dimensional representation of a dynamical system from a biased data set, even when employing strong biasing (i.e., the high bias-factor regime in the case of well-tempered metadynamics).

We underline that diffusion reweighting makes learning CVs from high-dimensional samples possible regardless of which conformational variable is biased to generate the data set. This extends the applicability of manifold learning methods to atomistic trajectories of any type (unbiased and biased) and makes it possible to learn CVs from a biased data set, where the sampling is faster and more evident than in an unbiased data set.

## 4 Conclusions

Nonlinear dimensionality reduction has been successfully applied to high-dimensional data without dynamical information. Dynamical data constitute a unique problem with different characteristics compared to generic data. Standard dimensionality reduction employed in analyzing dynamical data may result in a representation that does not contain dynamical information. This problem is even more pronounced in enhanced sampling, where we sample a biased probability distribution and additional assumptions on data structure have to be made. As such, manifold learning methods require a framework with several modifications that would allow for working on trajectories obtained from enhanced sampling simulations. In this work, we introduce such a framework.

The main result of our work is deriving the reweighting procedure for manifold learning methods that use transition probabilities for building low-dimensional embeddings. These advancements enable us to directly construct a low-dimensional representation of CVs from enhanced sampling simulations. We show how our approach can be leveraged to reconstruct slow CVs from enhanced sampling simulations even in high bias-factor regimes. Our framework can be further exploited in constructing a low-dimensional representation for dynamical systems using other manifold learning methods. For instance, it could be used in spectral embedding maps^29,35^ or stochastic neighbor embedding (e.g., *t*-sne).^30,31,63^ There are numerous stages at which such methods have scope for different algorithmic choices. Consequently, many possible algorithms can work within our framework.

An interesting direction for further research is to combine diffusion reweighting with a metric different from Euclidean distance, for instance, by considering a metric that enables introducing a lag time, as done in the case of kinetic and commute maps,^81−83^ a Mahalanobis kernel,^84,85^ or delay coordinates.^86^ Diffusion reweighting can be extended to yield intrinsic timescales directly from enhanced sampling simulations based on their relation to eigenvalues. We plan to take this road soon.

We underline that the presented diffusion reweighting can be used in any enhanced sampling method as the method can work with any functional form of the weights. For instance, tempering methods such as parallel tempering^87^ can be used, where the weights are given as e^–Δβ*U*^ for the difference in the inverse temperatures Δβ between the simulation temperature and the target temperature.

A point that requires further addressing is the selection of variables for a high-dimensional configuration space that carry enough information about the system dynamics to characterize a low-dimensional manifold. This issue is fundamental when using the configuration variables other than the microscopic coordinates. The configuration variables do not necessarily need to be optimal. We do not have to know whether all of the chosen configuration variables are relevant for the studied process; some of them may be spurious and thermodynamically meaningless. The primary assumption in selecting such configuration variables is that some are relevant and capture slower timescales of the studied process. This assumption can be validated by using a diffusion map (with reweighting if the samples are biased) to check if there is a clear separation of timescales and if the dynamics of the selected configuration variables is slower compared to other variables.^47,88^ Our framework can be used for this aim; therefore, we plan to investigate the effect of selecting the configuration variables on constructing the low-dimensional CVs in the future.

Our framework makes it possible to generate biased data sets that, given the construction of enhanced sampling methods, sample a larger conformational space than standard atomistic simulations and use such data to learn low-dimensional embeddings. If a data set entails many infrequent events, the low-dimensional representation is more prone to encode them quantitatively. Moreover, in the case of the reweighted stochastic embedding methods, which we cover here, the generated embeddings can be used for biasing in an iterative manner, for example, where we iterate between the learning and biasing phases. We believe that the accurate construction of the Markov transition probability matrix is a crucial element in implementing such an algorithm optimally without being restricted by kinetic bottlenecks (i.e., low-probability transition regions).

Overall, we expect that our approach to manifold learning from enhanced sampling simulations opens a variety of potential directions in studying metastable dynamics that can be explored.

## Appendix

### A Diffusion Reweighting

Consider a data set  where each sample **x**_*k*_ is high-dimensional, and the number of samples is given by *K* [eq 11].

A discrete probability distribution for a stochastic process with a discrete state space is given by

34

where ∑_*k*_*w*(**x**_*k*_) = 1. Assuming a Gaussian kernel *G*_ε_, we can account for the statistical weights to obtain the unbiased kernel density estimate [eq 15]

35

where the Dirac delta function δ(**x** – **x**_*l*_) leaves only the *l*-th terms from the integral. Then, up to a normalization constant, the diffusion-map kernel is given by

36

where the parameter α is called the anisotropic diffusion parameter. The normalization constant *d*(**x**) for eq 36 can be calculated similarly to eq 35

37

A Markov operator  acting on an auxiliary function *f*(**x**) can be written as

39

where *K* is known as a kernel of the Markov operator , and . Using the abovementioned definition, we can evaluate the Markov transition matrix *M*(**x**_*k*_, **x**_*l*_) by acting the Markov operator  on the function *f*(**x**_*k*_) using the anisotropic diffusion kernel [eq 36] as *K* in eq 39

40

which gives us the definition of the Markov transition matrix *M*.

By introducing a rescaled statistical weight

41

we can write *M*(**x**_*k*_, **x**_*l*_) as

42

Therefore, a general expression for the reweighting factor can be given as

43

where *w*_α_(**x**_*k*_) is canceled out in eq 42 during the normalization. Alternatively, we can express eq 43 using a biased density estimate ρ_*V*_(**x**_*k*_) = ∑_*l*_*G*_ε_(**x**_*k*_, **x**_*l*_) [eq 16]

44

which is similar to the standard reweighting formula. Using eq 44 and setting α = 1/2, we obtain

45

which concludes the derivation of eq 20.

### B Square-Root Approximation

Here, we want to derive eq 33 by considering approximations to the Markov transition matrix used in stke.^22^

As we discuss in Section 3.2, we want to obtain a format of the transition matrix similar to that of the square-root approximation to the Fokker–Planck operator. We start from the reweighting factor given by eq 31 and construct the following Markov transition matrix

46

where ρ(**x**_*k*_) is canceled out due to the normalization. By assuming that ε is sufficiently small, we can take the following approximation to the normalization constant of eq 32

47

where we approximate the average of local densities under the kernel density estimate by the density centered on **x**_*k*_. Then, eq 32 is

48

which gives us a relation similar to the square-root approximation of the infinitesimal generator of the Fokker–Planck operator^66−69^ [eq 33].

### C Gaussian Mixture for the Markov Transition Matrix *M*

Here, we describe a procedure used to automatically estimate bandwidths for a Gaussian mixture used in mrse. The procedure is similar to that used in *t*-sne, with the exemption of using a Gaussian mixture instead of a single Gaussian and expanding the procedure to account for the statistical weights. We follow a procedure outlined in ref (24).

We use a Gaussian mixture to represent the Markov transition matrix [eq 30]. Each Gaussian has a positive parameter set **ε** = {ε_*k*_}. We find the appropriate values of **ε** so that the Shannon–Gibbs entropy of each row of *M*(**x**_*k*_, **x**_*l*_) ≡ (*p*_*kl*_), *s*_*k*_ = −∑_*l*_*p*_*kl*_ log  *p*_*kl*_ is approximately equal to the number of neighbors *n*_*p*_ given as the logarithm of perplexity.^31^

Considering the weights of the exponential form, , where *V*_*k*_ is the relative bias potential at the *k*-th sample [eq 9], the entropy for the *k*-th row of the Markov transition matrix *M* has to be corrected by including the bias potential in comparison to that used in *t*-sne.^31^ The bias-free term is given by

50

and the correction term is

51

where the sum is the averaged bias potential with respect to the transition probabilities of the Markov transition matrix *M*.

Therefore, the optimization of **ε** is performed by finding such {ε_*k*_} so that it minimizes the difference between the Shannon–Gibbs entropy for the *k*-th row of *M* and the number of neighbors in a manifold

52

which can be solved using a binary search. After finding the set **ε** of bandwidths (each for a single row of *M*) for a perplexity value, we can calculate the Gaussian mixture representation of *M* as an average over *M* estimated for each selected perplexity. Perplexities for each *M* matrix can be also estimated automatically.

A detailed derivation and a discussion about the procedure outlined here can be found in ref (24).

### D Landmark Sampling: Selecting the Training Set

For stke, the training data set is selected using a geometric subsampling scheme that results in landmarks distributed uniformly. Specifically, the training data set is created such that min_*kl*_∥**x**_*k*_ – **x**_*l*_∥ ≥ *r*_c_, where *r*_c_ is a minimal pairwise distance, which modifies the level of sparsity for building the Markov transition matrix.

In mrse, we use weight-tempered random sampling in which the training data set is selected according to statistical weights. The statistical weights are scaled, *w*^1/τ^, where τ ≥ 1 is the tempering parameter, and samples are selected according to the scaled weights. It has been shown that in the limit of τ → ∞, we obtain the biased marginal probability, and for τ → 1, we recover the unbiased probability. A detailed discussion with a comparison to other landmark sampling algorithms is provided in ref (24).

### E Parameters for Reweighted Stochastic Embedding

We show a summary of the reweighted stochastic embedding methods and parameters in Table 1. Note that many parameters for the reweighted stochastic embedding methods are set as those in refs (22) and (24).

**Table 1:** Summary of Reweighted Stochastic Embedding Methods Used in (Section 3.2): mrse and stke^a^

	mrse	stke
reweighting factor *r*(**x**_*k*_, **x**_*l*_)	[eq 29]	[eq 31]
high-dim. prob. *M*(**x**_*k*_, **x**_*l*_)	Gaussian mixture [eq 30] for perplexities ∈ {256, 128, 64, 32}	Gaussian [eq 32] with ε = 0.12
low-dim. prob. *Q*(**z**_*k*_, **z**_*l*_)	*t*-distribution [eq 26]	Gaussian with ε = 0.12
landmark sampling	weight-tempered random sampling for τ = 3 and 5000 landmarks	minimal pairwise distance for *r*_*c*_ = 1.2 and 97000 landmarks
activation functions	ReLU (3 layers)	hyperbolic tangent (3 layers)
optimizer	Adam (μ = 0.001, β_1_ = 0.9, β_2_ = 0.999)	Adam (μ = 0.001, β_1_ = 0.9, β_2_ = 0.999)
batch size	1000	256

a Labels: reweighting factor *r*(**x**_*k*_, **x**_*l*_), high-dimensional Markov transition matrix *M*(**x**_*k*_, **x**_*l*_), and low-dimensional Markov transition matrix *Q*(**z**_*k*_, **z**_*l*_).

## Supporting Information Available

Details of data sets: one-dimensional potential, alanine dipeptide, and chignolin; additional figures: density of samples along the coordinate *x* for the one-dimensional potential; and diffusion coordinates and effective timescales for alanine dipeptide (PDF)
