# Supplementary material for: Reweighted Manifold Learning of Collective Variables from Enhanced Sampling Simulations
Source: J Chem Theory Comput. 2022 Nov 11;18(12):7179–92. doi: 10.1021/acs.jctc.2c00873 (PMC9753586; doi:10.1021/acs.jctc.2c00873)
Supplement: Supplementary file 1 — ct2c00873_si_001.pdf [file ct2c00873_si_001.pdf]

Supporting Information: Reweighted Manifold Learning of Collective Variables from Enhanced Sampling Simulations

Jakub Rydzewski,[*,†] Ming Chen,[‡] Tushar K. Ghosh,[‡] and Omar Valsson[¶]

†*Institute of Physics, Faculty of Physics, Astronomy and Informatics, Nicolaus Copernicus University, Grudziadzka 5, 87-100 Toruń, Poland*

‡*Department of Chemistry, Purdue University, West Lafayette, Indiana 47907, USA.*

¶*Department of Chemistry, University of North Texas, Denton, Texas 76201, USA*

E-mail: jr@fizyka.umk.pl

S1 Data Sets

A 1D Potential

We perform the simulation of a single particle moving on a simple one-dimensional potential given by:

$$U(x) = W(x) + \sum_{k=1} \alpha_k \exp\left(-\frac{1}{\sigma_k}\|x - \xi_k\|^2\right), \tag{S1}$$

where $\boldsymbol{\alpha} = (-100, -60, -50)$, $\boldsymbol{\xi} = (2, 5, 8)$, $\boldsymbol{\sigma} = (1, 3, 1)$, $W(x) = 2\|x - \xi_2\|^2$, and $U(x)$ is shifted so its minimum is at zero. We employ rescaled units such that $k_{\mathrm{B}}T = 1$. The free-energy profile is given directly by the potential $U(x) \equiv F(x)$. We use the `pesmd` code from PLUMED[S1,S2] to simulate the system at a temperature of $T = 1$ using a Langevin thermostat[S3] with a friction coefficient of 10 and a time step of 0.005. At this temperature,

the potential has a barrier of around 50 $k_\mathrm{B}T$. To generate a biased data set, we perform the well-tempered metadynamics simulation using x as a CV. We employ a bias factor of 10, an initial initial Gaussian height of 2, a Gaussian width of 0.5, and deposit the bias every 100 steps. We calculate $c(t)$ every time a Gaussian is deposited along x. We run the simulation for 2×10^7 steps. For the diffusion map, we use $\varepsilon = 0.1$.

B Alanine Dipeptide

We perform a 100-ns alanine dipeptide (Ace-Ala-Nme) simulation using the GROMACS 2019.2 code[S4] patched with a development version of the PLUMED plugin.[S1,S2] We use the Amber99-SB force field,[S5] and a time step of 2 fs. We perform the simulations in the canonical ensemble using the stochastic velocity rescaling thermostat[S6] with a relaxation time of 0.1 fs. We constrain hydrogen bonds using LINCS.[S7] The simulations are performed in vacuum without periodic boundary conditions and with no cut-offs for electrostatic and non-bonded van der Waals interactions. To generate a biased data set, we perform the well-tempered metadynamics simulations at 300 K using the backbone dihedral angles Φ and Ψ as CVs and employ a bias factor of 5. We use an initial Gaussian height of 1.2 kJ/mol, a Gaussian width of 0.2 rad for both CVs, and deposit Gaussians every 1 ps. We calculate $c(t)$ every time a Gaussian is added. We run the simulation for 100 ns. We skip the first 20 ns of the runs to ensure that we avoid the period at the beginning of the simulations where the weights might be unreliable due to rapid changes in the bias potential. For the diffusion-map embeddings, we use 45 heavy atoms pairwise distances as input sampled every 50 ps which given a data set of dimensions 1600×45. The diffusion coordinates are constructed using $\varepsilon = 0.078$ that is computed as the median of the pairwise distances.

C Chignolin

We perform a 1-μs chignolin (CNL025) simulation starting from an NMR structure[S8] using the GROMACS 2019.2 code[S4] patched with a development version of the PLUMED plugin.[S1,S2]

We use the CHARMM27 force field,[S9] and a time step of 2 fs. We perform the simulations in the canonical ensemble using the stochastic velocity rescaling thermostat[S6] with a relaxation time of 0.1 fs. We constrain hydrogen bonds using LINCS.[S7] The simulations are performed in solution (TIP3P) with periodic boundary conditions and cut-offs for electrostatic and non-bonded van der Waals interactions of 1 nm. To generate a biased data set, we perform the well-tempered metadynamics simulations at 340 K using the distance between the CNL025 Cα atoms of Y1 and Y10 and the radious of gyration as CVs and employ a bias factor of 20. We use an initial Gaussian height of 2 kJ/mol, Gaussian widths of 0.1 nm for the CVs, and deposit Gaussians every 1 ps. We calculate $c(t)$ every 500 added Gaussians. We skip the first 20 ns of the runs to avoid unreliable statistical weights.

S2 Additional Figures

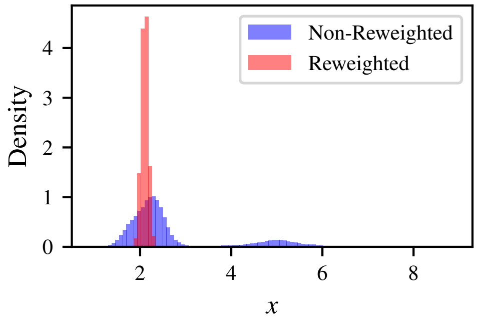

Figure S1: Density of samples along the coordinate x for the one-dimensional potential [Eq. (S1)] constructed without reweighting (blue) and with reweighing (red). The samples are generated using well-tempered metadynamics with a bias factor of 10.

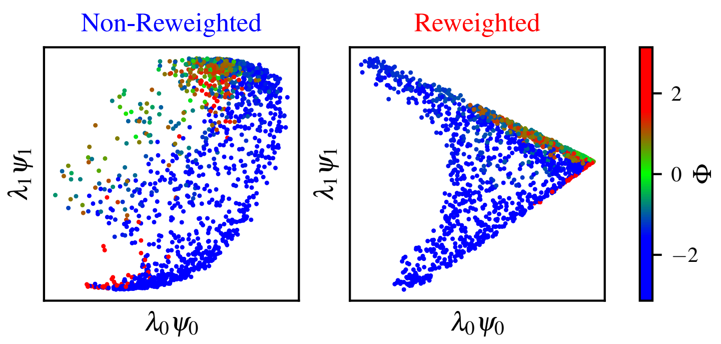

Figure S2: Diffusion coordinates $\lambda_0\psi_0$ vs. $\lambda_1\psi_1$ for the non-reweighted (blue label) and reweighted (red label) transition probability matrices for the alanine dipeptide system. Each sample corresponds to the value of the Φ dihedral angle.

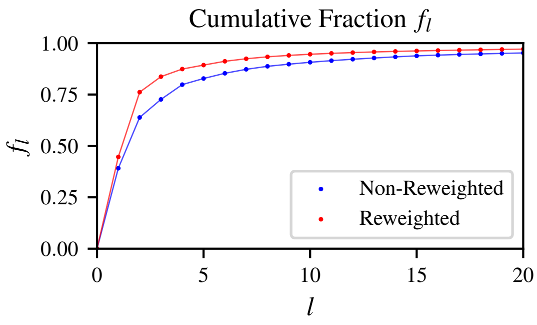

Figure S3: Cumulative fraction f_l calculated for the non-reweighted (blue) and reweighted (red) transition probability matrices for respective eigenvalues. The cumulative fraction of eigenvalues $\{\lambda_l\}$ is calculated as $f_l = \sum_{i=0}^{l} \lambda_i / \sum_{k=0}^{N} \lambda_k$, where N corresponds to the total number of eigenvalues.

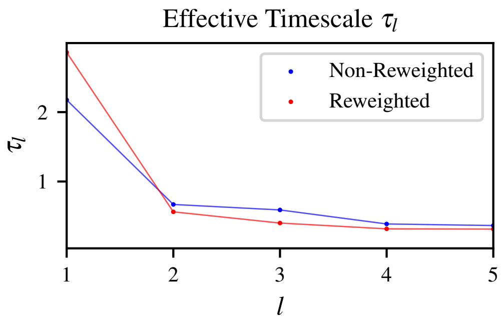

Figure S4: Effective timescale τ_l (unitless) calculated for the non-reweighted (blue) and reweighted (red) transition probability matrices for respective eigenvalues. The effective timescale of eigenvalues $\{\lambda_l\}$ is calculated as $\tau_l = -\frac{1}{\log \lambda_l}$.

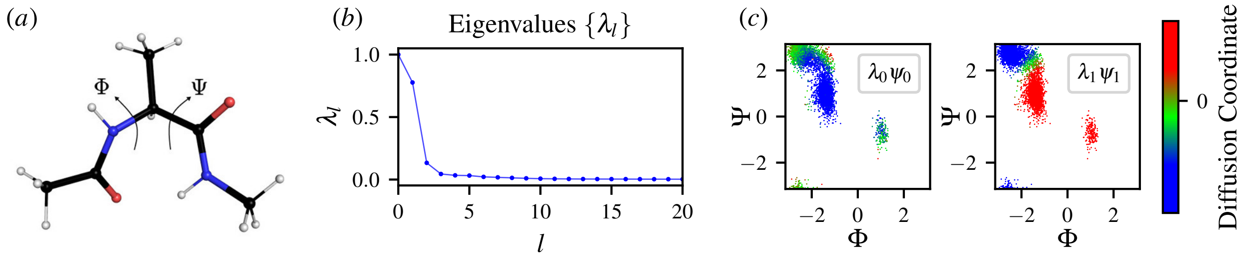

Figure S5: Diffusion coordinates and eigenvalue spectrum for the unbiased parralel-tempering simulation of alanine dipeptide. The results are obtained using the equilibrium replica at 300 K.

References

(S1) G. A. Tribello, M. Bonomi, D. Branduardi, C. Camilloni, and G. Bussi, *PLUMED 2: New Feathers for an Old Bird,* Comp. Phys. Commun. **185**, 604–613 (2014).

(S2) PLUMED Consortium, *Promoting Transparency and Reproducibility in Enhanced Molecular Simulations,* Nat. Methods **16**, 670–673 (2019).

(S3) G. Bussi and M. Parrinello, *Accurate Sampling using Langevin Dynamics,* Phys. Rev. E **75**, 056707 (2007).

(S4) M. J. Abraham, T. Murtola, R. Schulz, S. Páll, J. C. Smith, B. Hess, and E. Lindahl, *GROMACS: High Performance Molecular Simulations through Multi-Level Parallelism from Laptops to Supercomputers,* SoftwareX **1–2**, 19–25 (2015).

(S5) V. Hornak, R. Abel, A. Okur, B. Strockbine, A. Roitberg, and C. Simmerling, *Comparison of Multiple Amber Force Fields and Development of Improved Protein Backbone Parameters,* Proteins **65**, 712–725 (2006).

(S6) G. Bussi, D. Donadio, and M. Parrinello, *Canonical Sampling through Velocity Rescaling,* J. Chem. Phys. **126**, 014101 (2007).

(S7) B. Hess, *P-LINCS: A Parallel Linear Constraint Solver for Molecular Simulation,* J. Chem. Theory Comput. **4**, 116–122 (2008).

(S8) S. Honda, T. Akiba, Y. S. Kato, Y. Sawada, M. Sekijima, M. Ishimura, A. Ooishi, H. Watanabe, T. Odahara, and K. Harata, *Crystal Structure of a Ten-Amino Acid Protein,* J. Am. Chem. Soc. **130**, 15327–15331 (2008).

(S9) A. D. Mackerell Jr, M. Feig, and C. L. Brooks III, *Extending the Treatment of Backbone Energetics in Protein Force Fields: Limitations of Gas-Phase Quantum Mechanics in Reproducing Protein Conformational Distributions in Molecular Dynamics Simulations,* J. Comput. Chem. **25**, 1400–1415 (2004).
